# Env-pseudoviruses based on the HIV-1 genetic variant circulating in Siberia

**DOI:** 10.18699/vjgb-25-63

**Published:** 2025-07

**Authors:** N.B. Rudometova, A.A. Fando, D.N. Shcherbakov, B.N. Zaitsev, A.P. Rudometov, L.I. Karpenko

**Affiliations:** State Research Center Center of Virology and Biotechnology “Vector” Rospotrebnadzor, Koltsovo, Novosibirsk region, Russia; State Research Center Center of Virology and Biotechnology “Vector” Rospotrebnadzor, Koltsovo, Novosibirsk region, Russia; State Research Center Center of Virology and Biotechnology “Vector” Rospotrebnadzor, Koltsovo, Novosibirsk region, Russia; State Research Center Center of Virology and Biotechnology “Vector” Rospotrebnadzor, Koltsovo, Novosibirsk region, Russia; State Research Center Center of Virology and Biotechnology “Vector” Rospotrebnadzor, Koltsovo, Novosibirsk region, Russia; State Research Center Center of Virology and Biotechnology “Vector” Rospotrebnadzor, Koltsovo, Novosibirsk region, Russia

**Keywords:** HIV-1, CRF63_02A6, Env-pseudoviruses, bnAbs, virus neutralization assay, ВИЧ-1, CRF63_02A6, Env-псевдовирусы, bnAb, анализ вирусной нейтрализации

## Abstract

Despite numerous efforts of the global community, it is still not possible to stop the HIV/AIDS pandemic. To stop the spread of the virus, an effective preventive vaccine is needed, as well as the search for new antiviral agents. In order to be able to quickly and adequately evaluate the developed vaccine constructs, characterize HIV-specific antibodies and potential drugs, a reliable testing method is needed. In this regard, pseudotype neutralization assays using a panel of Env-pseudoviruses of different HIV-1 subtypes has proven itself well. Currently, separate panels of Env-pseudoviruses of the main genetic subtypes of HIV-1 (A, B, C and a number CRFs) have been created. These panels are necessary to obtain standardized data sets that can be used to rank the effectiveness of the vaccine and identify promising candidates for further study. Currently, the HIV-1 subtype A6 dominates in the European part of Russia, and the recombinant form CRF63_02A6, which has currently been detected in more than 80 % of new HIV-1 cases in Siberia, dominates in Siberia. The aim of this work was to expand and characterize the collection of Env-pseudoviruses obtained on the basis of the recombinant form CRF63_02A6 of HIV-1 circulating in Siberia. In this study, two new variants of Env-pseudoviruses based on CRF63_02A6 of HIV-1 were obtained, characterized, and included in our collection. At present, the collection includes 13 Env-pseudoviruses that are CCR5-tropic. Phylogenetic analysis of the full-length nucleotide sequences of the env gene confirmed that all 13 pseudoviruses cluster with the reference sequences of the recombinant form CRF63_02A6. The Env-pseudoviruses were characterized using broadly neutralizing antibodies (bnAbs) targeting different regions of vulnerability of HIV-1 located on the surface of Env glycoprotein complexes. It was shown that the Env-pseudoviruses are sensitive to neutralization by bnAbs VRC01 and 10E8; moderately sensitive to neutralization by bnAbs PG9 and PGT126; and resistant to neutralization by antibodies 2G12 and 2F5. The resulting collection is an important addition to the existing panels of pseudoviruses against other HIV-1 subtypes in the world.

## Abstract (ru)

Несмотря на многочисленные усилия мирового сообщества, остановить пандемию ВИЧ/СПИД пока не удается. Чтобы остановить распространение вируса, требуются эффективная профилактическая вакцина, а также поиск новых противовирусных агентов. Для того чтобы иметь возможность быстро и адекватно оценивать разрабатываемые вакцинные конструкции, характеризовать ВИЧ-специфические антитела и потенциальные лекарственные препараты, необходим надежный метод тестирования. В этом аспекте хорошо зарекомендовал себя анализ нейтрализации вирусного псевдотипа с использованием панели Env-псевдовирусов разных субтипов ВИЧ-1. В настоящее время созданы отдельные панели псевдовирусов основных генетических подтипов ВИЧ-1: А, В, С и ряда CRF. Эти панели необходимы для получения стандартизированных наборов данных, которые могут быть использованы для ранжирования эффективности вакцины и выявления перспективных кандидатов для дальнейшего исследования. На сегодняшний день в европейской части России доминирует субтип А6 ВИЧ-1, а в Сибири – рекомбинантная форма CRF63_02A6, которая обнаруживается более чем в 80 % новых случаев ВИЧ-1 в Сибири. Цель работы состояла в расширении и характеристике коллекции Env- псевдовирусов, полученных на основе рекомбинантной формы CRF63_02A6 ВИЧ-1, циркулирующей в регионах Сибири. В настоящем исследовании получены и охарактеризованы два новых варианта Env-псевдовирусов на основе CRF63_02A6 ВИЧ-1, которые вошли в нашу коллекцию. Коллекция включает 13 Env-псевдовирусов, являющихся CCR5-тропными. Филогенетический анализ полноразмерных нуклеотидных последовательностей гена env подтвердил, что все 13 псевдовирусов кластеризуются с референсными последовательностями рекомбинантной формы CRF63_02A6. Env-псевдовирусы были охарактеризованы с помощью широко нейтрализующих антител (bnAb), нацеленных на разные регионы уязвимости ВИЧ-1, расположенных на поверхности гликопротеиновых комплексов Env. Показано, что Env-псевдовирусы чувствительны к нейтрализации bnAb VRC01 и 10E8; умеренно чувствительны к нейтрализации bnAb PG9 и PGT126; и устойчивы к нейтрализации антителами 2G12 и 2F5. Полученная коллекция – важное дополнение к существующим в мире панелям псевдовирусов против других подтипов ВИЧ-1.

## Introduction

Human immunodeficiency virus type 1 (HIV-1) remains a major global public health problem. Despite the successes of antiretroviral therapy (ART), it is still not possible to stop the spread of HIV. According to WHO, about 1.2 million people are infected with HIV-1 annually (HIV statistics, globally and by WHO region, 2024). This is largely due to the fact that the use of drugs to treat HIV infection is accompanied by the emergence of resistance mutations in the virus (HIV drug resistance: brief report, 2024). As a result, the development of more effective drugs is required. Vaccination of the population could provide reliable protection against HIV/AIDS, but, unfortunately, an effective vaccine has not yet been created (Levy, 2024). Developing a vaccine against HIV-1 is a very difficult task due to the high variability of the virus and its ability to integrate into the human genome. Research in this area is currently focused on the development of immunogens capable of inducing antibodies that neutralize a broad range of HIV-1 isolates (bnAbs) (Trkola, Moore, 2024).

In order to be able to rapidly and reliably evaluate vaccine constructs under development, characterize HIV-specific antibodies and search for potential drugs (entry inhibitors), a reliable testing method is needed. Env-pseudovirus technology has proven itself to be the best in this regard. Neutralizing activity assays using pseudoviruses have several advantages over traditional replicating virus systems in peripheral blood mononuclear cell cultures. First, pseudoviruses are incapable of replication and can be safely handled outside an expensive biosafety level 3 laboratory. Second, neutralization or inhibition assays can be performed using a continuous cell line, thereby reducing the need for primary donor cells. Together, these elements ensure the accuracy, reproducibility and standardization of pseudovirus-based assays (Rudometova et al., 2022a).

Currently, separate panels of pseudoviruses of the main HIV-1 genetic subtypes (A, B, C and a number of CRFs) have been created (Li et al., 2005; Hraber et al., 2017; Wang et al., 2018; Stefic et al., 2019). These panels are needed to obtain standardized data sets that can be used to rank vaccine efficacy and identify promising candidates for further study (de Camp et al., 2014).

It should be noted that in the Russian Federation, the genetic diversity of HIV-1 has regional specificity (Antonova et al., 2023). In the European part of Russia, the HIV-1 subtype A6 dominates (Antonova et al., 2023; Kuznetsova et al., 2023), and in the Siberian region, the HIV-1 recombinant form CRF63_02A6 (Maksimenko et al., 2020; Rudometova et al., 2021), which is currently detected in more than 80 % of new HIV-1 cases in Siberia (Sivay et al., 2022). Some data indicate that HIV-1 CRF63_02A6 is more infectious and may have higher replicative activity than other subtypes, which may lead to an increase in the number of people infected with this variant of the virus (Bogacheva et al., 2017). In connection with the above, there is a need to develop a separate collection of pseudoviruses for HIV-1 CRF63_02A6.

Previously, we described the production of a number of HIV-1 CRF63_02A6 Env-pseudoviruses (Rudometova et al., 2022b). However, to ensure the relevance of the collection, ongoing work is required to obtain new pseudoviruses based on circulating HIV-1 strains.

The aim of this work was to expand and characterize the collection of Env-pseudoviruses obtained based on the recombinant form CRF63_02A6 of HIV-1 circulating in the Siberian region of Russia.

## Materials and methods

For the study, 63 serum samples taken from HIV-infected blood donors from the Kemerovo Region and the Altai Republic were selected. They were provided by regional AIDS centers in accordance with the decisions made by the ethical committees of the above-mentioned organizations. Each sample was assigned an anonymous number in accordance with the requirements of the ethical standards of the Russian Federation. Then, RNA was isolated from individual serum using the MAGNO-Sorb reagent kit (Amplisens, Russia) according to the manufacturer’s recommendations. The isolated RNA samples were stored at –80 °C.

To obtain amplified variants of the HIV-1 env gene, a reverse transcription – PCR reaction (RT-PCR) was performed using the Env_S-1 and Env_02A_AS_1 primers (5′-TTGG GTGTCAACATAGCAGAATAGG-3′ and 5′-CCTGTGGC CTGACTGGAAAGC-3′) and the SuperScript™ IV One-Step RT-PCR System reagent kit (Invitrogen, USA) according to the manufacturer’s recommendations. After RT-PCR, the amplification products were analyzed by electrophoresis in a 1 % agarose gel.

Amplified variants of the HIV-1 env gene were cloned into the commercial expression vector pcDNA3.1/V5-His TOPO TA (Invitrogen). Transformation of chemically competent Escherichia coli strain Stbl3 cells (Thermo Fisher Scientific, USA) was performed using the heat shock method (Chang et al., 2017) and clones carrying a plasmid with the integrated env gene were selected.

To produce Env-pseudoviruses, HEK293 cell culture was transfected with the constructed recombinant pEnv plasmids together with the pSG3Δenv core plasmid (NIH Reagent Program) using Lipofectamine 3000 (Invitrogen) in a 6-well plate format. Forty-eight hours after transfection, pseudovirus particles were collected by filtering the culture medium through a 0.45-micron filter; concentrated in 20 % sucrose solution for 3 hours at 25,000 rpm; aliquots of 1 ml were made and stored at –80 °C.

Electron microscopic images were obtained by the negative contrast method using a JEM-1400 electron microscope with an accelerating voltage of 80 kV, at magnifications from 10,000 to 80,000x.

The functional activity of pseudoviruses was determined using the TZM-bl cell line (NIH Reagent Program, USA) according to the method described previously (Revilla et al., 2011). Pseudovirus clones were considered functional and suitable for further work if the luminescence level of TZM- bl cells carrying these clones was at least 50 times higher than the luminescence level of cells without the addition of pseudovirus particles.

The nucleotide sequences of the amplified variants of the HIV-1 env gene were determined by sequencing using the Sanger method (Genomics Collective Use Center, Institute of Chemical Biology and Fundamental Medicine, Siberian Branch of the Russian Academy of Sciences, Novosibirsk). The resulting sequencing chromatograms were analyzed using the BioEdit program.

The subtype of HIV-1 env gene variants was determined using the HIV BLAST online resource (https://www.hiv.lanl. gov/content/sequence/BASIC_BLAST/basic_blast.html). Phylogenetic tree construction was performed using IQ-TREE on the portal https://www.hiv.lanl.gov/content/sequence/ IQTREE/iqtree.html. Reference sequences from the Los Alamos HIV Sequence Database (http://www.hiv.lanl.gov/) were used to construct phylogenetic trees. Tropism of the obtained HIV-1 Env pseudoviruses to the coreceptor (CCR5 or CXCR4) was determined by the nucleotide sequence of the V3 loop using the geno2pheno online program (http:// coreceptor.geno2pheno.org/).

Neutralization assay was performed according to the method described previously (Revilla et al., 2011). In the work, monoclonal bnAbs to HIV-1 were used, targeting different regions of vulnerability on the surface of trimeric gp120/gp41 complexes – 2G12, VRC01, PG9, PGT126, 2F5, 10E8 (NIH HIV Reagent Program). Samples were tested in duplicate; the experiment was repeated twice. Statistical data processing and IC50 calculation were performed using the GraphPad Prism 9 software.

## Results

**Obtaining new HIV-1 Env-pseudoviruses variants**

As a result of the study, five full-length variants of the env gene were amplified using RT-PCR and cloned into an expression plasmid vector. For each env gene variant, one to six genetic constructs with an insert were obtained and used for co-transfection.

**Fig. 1.**
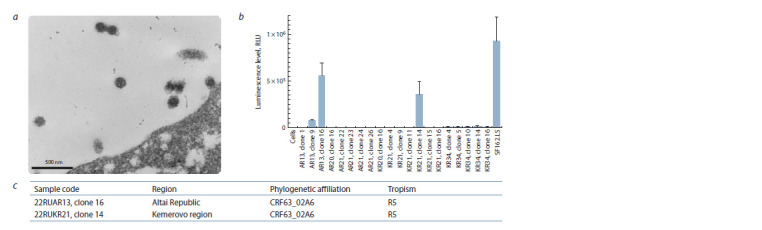
Characteristics of HIV-1 Env-pseudoviruses. а – electron micrograph of HEK293 cells after transfection with budded pseudoviral particles of the recombinant form CRF63_02A6; b – functional activity of the obtained variants of HIV-1 Env-pseudoviruses (data are presented as mean ± standard deviation); Env-pseudovirus SF162.LS subtype B from the international reference panel of pseudoviruses was used as a positive control; c – characteristics of functionally active HIV-1 Env-pseudoviruses.

Next, we produced Env-pseudoviruses and determined their functional activity. The formation of pseudovirus particles was recorded using electron microscopy (Fig. 1, a). Functional analysis of the obtained pseudovirus variants showed that the assembly of functionally active pseudovirus particles occurs for two pseudovirus variants: 22RUAR13 (clone 16) and 22RUKR21 (clone 14) (Fig. 1, b). The luminescence level of the cells carrying these clones was more than 150 times higher than the luminescence level of pure cells. Sequencing data and phylogenetic analysis confirmed that the functional Envpseudoviruses belong to the recombinant form CRF63_02A6 and are CCR5-tropic (Fig. 1, c).

**Characteristics of the HIV-1 CRF63_02A6 Env-pseudoviruses collection**

**Fig. 2.**
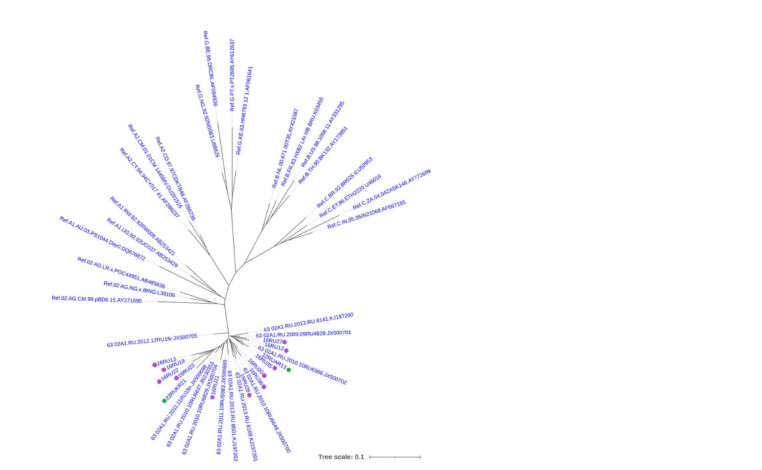
Maximum likelihood phylogenetic tree of the env gene of HIV-1 pseudoviruses (visualized using the iTOL online tool). Green dots mark the env genes of clones 22RUAR13 (clone 16) and 22RUKR21 (clone 14). Purple dots mark the env genes of pseudoviruses obtained earlier (Rudometova et al., 2022b).

Previously, we obtained 11 Env-pseudoviruses belonging to the recombinant form CRF63_02A6 (Rudometova et al., 2022b). In this work, we added two new pseudoviruses to the collection and decided to conduct a more detailed characterization of it. A phylogenetic analysis of the full-length nucleotide sequences of the env gene was carried out, which confirmed that all 13 pseudoviruses cluster with the reference sequences of the recombinant form CRF63_02A6 (Fig. 2). From the presented data (Fig. 2), it is evident that a certain genetic heterogeneity is observed due to the presence of differences in the nucleotide sequences of the env genes of the pseudoviruses included in the collection.

**Neutralization phenotype of the HIV-1 CRF63_02A6 Env-pseudoviruses collection**

**Fig. 3.**
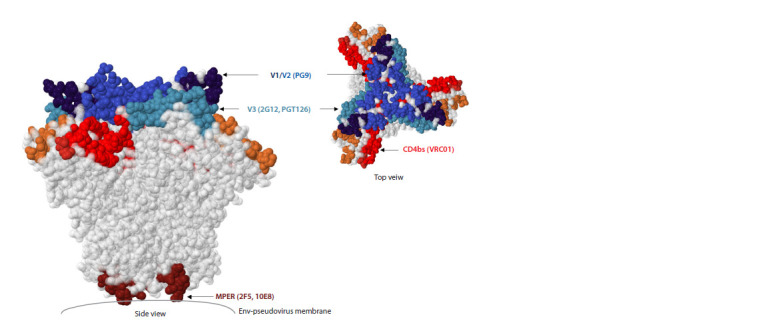
Sites of vulnerability of broadly neutralizing antibodies in the Env trimer model (PDB: 4ZMJ). The HIV-1 Env trimer model was visualized using the HIV 3D Structure Viewer program. The designations of broadly neutralizing antibodies (bnAbs) binding to the vulnerability sites of the virus and used in this work are given in brackets.

Env-pseudoviruses with functional activity were tested in a pseudovirus neutralization assay with antibodies capable of neutralizing a broad range of HIV-1 isolates (bnAbs). bnAbs recognizing the main sites of HIV-1 vulnerability on the gp120/gp41 trimeric complex were selected for analysis (Fig. 3): bnAbs VRC01 – the virus binding to the CD4 cellular receptor; bnAbs PG9 – the structure of the V1/V2 variable loops at the top of the trimer; bnAbs 2G12 and PGT126 – the V3 loop region in the glycan complex; bnAbs 2F5 and 10E8 – the membrane-proximal external region of gp41 (MPER) (Walsh, Seaman, 2021; Thavarajah et al., 2024).

**Fig. 4.**
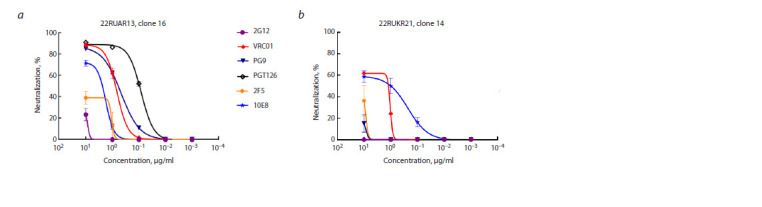
Neutralizing activity of bnAbs against Env-pseudoviruses 22RUAR13, clone 16 (a) and 22RUKR21, clone 14 (b).

As an example, Fig. 4 shows typical neutralization curves of Env-pseudoviruses by monoclonal broadly neutralizing antibodies for the new variants 22RUAR13 (clone 16) and 22RUKR21 (clone 14).

According to the obtained data (Fig. 4), Env-pseudovirus 22RUAR13 (clone 16) was highly sensitive to neutralization by bnAbs VRC01, PG9 and PGT126, showed moderate sensitivity to neutralization by antibody 10E8, and was resistant to neutralization by antibodies 2G12 and 2F5. At the same time, pseudovirus 22RUKR21 (clone 14) demonstrated moderate sensitivity to neutralization by bnAbs VRC01 and 10E8 and was resistant to neutralization by antibodies 2G12, PG9, 2F5 and PGT126

Table shows the IC50 values determined for bnAbs against our entire collection of HIV-1 CRF63_02A6 Env-pseudoviruses, including 22RUAR13 and 22RUKR21. Comparative analysis of IC50 showed that Env-pseudoviruses of this collection have rather heterogeneous sensitivity to neutralization by bnAbs (see the Table), which is probably due to their antigenic differences (Supplementary Material)3. As can be seen from Table, HIV-1 CRF63_02A6 Env-pseudoviruses demonstrate high sensitivity to neutralization by bnAbs VRC01 and 10E8

**Table 1.**
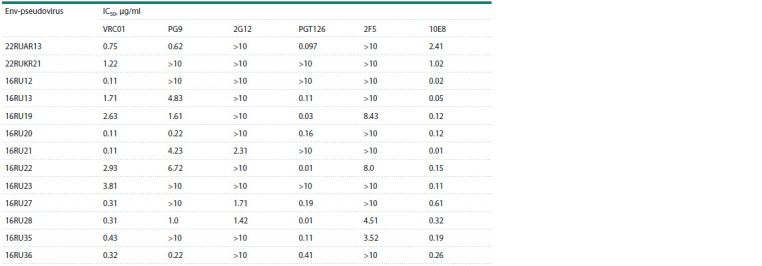
IC50 values determined for bnAbs against HIV-1 CRF63_02A6 Env-pseudoviruses


Supplementary Materials are available in the online version of the paper: https://vavilov.elpub.ru/jour/manager/files/Suppl_Rudomet_Engl_29_4.pdf


These antibodies are known to have high neutralizing activity against other HIV-1 subtypes, including A, B, C, recombinant forms CRF02_AG and CRF01_AE (Wu et al., 2010; Huang et al., 2012; Hraber et al., 2017; Wang et al., 2018; Stefic et al., 2019; Wieczorek et al., 2024). In contrast, Env-pseudoviruses demonstrated moderate or even low sensitivity to neutralization against 2F5, PG9, 2G12 and PGT126 antibodies. This is likely due to the fact that bnAbs PG9, 2G12 and PGT126 recognize conformational epitopes in complex with glycans, which can interfere with efficient binding and, as a consequence, neutralization of the virus (Sanders et al., 2002; Doores, Burton, 2010; Krumm et al., 2016).

## Discussion

Recombination and mutational variability of HIV-1 have a significant impact on the changes in circulating HIV-1 viruses in Russia. As a result, not only the emergence of new recombinant forms of HIV is observed, but also their spread with the formation of new, different phylogenetic clusters of HIV-1. Such changes in the genetic characteristics of modern HIV-1 must be considered by developers of antiretroviral drugs and vaccines (Rashid et al., 2022; Nair et al., 2024). This requires verification tools, which include collections of Env-pseudoviruses of different HIV-1 subtypes (deCamp et al., 2014).

In this study, two new variants of HIV-1 Env-pseudoviruses were obtained and characterized based on genetic variants circulating in the Siberian Federal District. Phylogenetic analysis showed that both variants belong to the recombinant form CRF63_02A6 and are CCR5-tropic (Fig. 1, c; Fig. 2). The obtained variants of HIV-1 Env-pseudoviruses expanded the existing collection. Thus, our collection currently includes 13 Env-pseudoviruses belonging to the recombinant form CRF63_02A6. Env-pseudoviruses were characterized using bnAbs targeting different regions of HIV-1 vulnerability located on the surface of Env glycoprotein complexes. The analysis results showed that the Env-pseudoviruses included in the collection exhibit high sensitivity to bnAbs VRC01 and 10E8; moderate sensitivity to bnAbs PG9 and PGT126 and resistance to antibodies 2G12 and 2F5 (see the Table).

The advantage of this collection is that it is relatively representative, as it includes pseudoviruses that differ in both genetic diversity and sensitivity to bnAbs. According to the P. Hraber et al. (2017), a collection of 12 pseudoviruses of a certain HIV-1 subtype may be sufficient for the initial assessment of the efficacy of vaccine candidates.

Due to the fact that the recombinant form of CRF63_02A6 HIV-1 currently accounts for about 80 % in the Siberia and continues to actively spread (Sivay et al., 2022), the developed collection is an important addition to existing collections in the world against other HIV-1 subtypes.

## Conclusion

This work supplements and characterizes the collection of Env-pseudoviruses of HIV-1 recombinant form CRF63_02A6, which will allow to carry out a complex of scientific and applied works: to study the antiviral activity of created chemotherapeutic drugs and to evaluate the effectiveness of vaccines against HIV-1; to study the breadth and spectrum of neutralizing properties of monoclonal broadly neutralizing antibodies and to search for new broadly neutralizing antibodies by screening the neutralizing properties of blood sera of HIV-1 infected people, as well as to study the subtype-specific features of circulating genetic variants of HIV-1.

## Conflict of interest

The authors declare no conflict of interest.
